# Complete chloroplast genome of *Castanopsis sclerophylla* (Lindl.) Schott: Genome structure and comparative and phylogenetic analysis

**DOI:** 10.1371/journal.pone.0212325

**Published:** 2019-07-30

**Authors:** Xuemin Ye, Dongnan Hu, Yangping Guo, Rongxi Sun

**Affiliations:** Jiangxi Provincial Key Laboratory of Silviculture, College of Forestry, Jiangxi Agricultural University, Nanchang, China; Saint Mary's University, CANADA

## Abstract

*Castanopsis sclerophylla* (Lindl.) Schott is an important species of evergreen broad-leaved tree in subtropical areas and has high ecological and economic value. However, there are few studies on its chloroplast genome. In this study, the complete chloroplast genome sequence of *C*. *sclerophylla* was determined using the Illumina HiSeq 2500 platform. The complete chloroplast genome of *C*. *sclerophylla* is 160,497 bp long, including a pair of inverted repeat (IR) regions (25,675 bp) separated by a large single-copy (LSC) region of 90,255 bp and a small single-copy (SSC) region of 18,892 bp. The overall GC content of the chloroplast genome is 36.82%. A total of 131 genes were found; of these, 111 genes are unique and annotated, including 79 protein-coding genes, 27 transfer RNA genes (tRNAs), and four ribosomal RNA genes (rRNAs). Twenty-one genes were found to be duplicated in the IR regions. Comparative analysis indicated that IR contraction might be the reason for the smaller chloroplast genome of *C*. *sclerophylla* compared to three congeneric species. Sequence analysis indicated that the LSC and SSC regions are more divergent than IR regions within *Castanopsis*; furthermore, greater divergence was found in noncoding regions than in coding regions. The maximum likelihood phylogenetic analysis showed that four species of the genus *Castanopsis* form a monophyletic clade and that *C*. *sclerophylla* is closely related to *Castanopsis hainanensis* with strong bootstrap values. These results not only provide a basic understanding of *Castanopsis* chloroplast genomes, but also illuminate *Castanopsis* species evolution within the Fagaceae family. Furthermore, these findings will be valuable for future studies of genetic diversity and enhance our understanding of the phylogenetic evolution of *Castanopsis*.

## Introduction

*Castanopsis* (Lindl.) Schott. is a monoecious, broad-leaved tree of the genus *Castanopsis* belonging to the Fagaceae family. The genus contains approximately 120 known species, of which 58 are native and 30 are endemic to China. However, *C*. *sclerophylla* is widely distributed in East and South Asia, and the tree has been introduced to North America[1, 2]. In China, *C*. *sclerophylla* is a canopy tree widely distributed in subtropical evergreen forests[3]. Its fruit and wood are valuable, and it is regarded as a landscape and ornamental tree because of its glossy evergreen leaves and abundant white flowers[4]. In Jiangxi Province, the fruit of this tree has been used to make special foods such as sheet jelly, bean curd, and bean vermicelli[5]. Previous studies have mainly focused on the natural regeneration[6], biomass[7], morphology[8], chemotaxonomy[9], and genetic diversity[1, 10] of this species. However, because of the increasing economic value of the tree, natural trees are severely destroyed by humans, and their number is decreasing due to the slow growth rate of this species. As a result, the distribution of *C*. *sclerophylla* is severely fragmented and constantly threatened, requiring urgent conservation and restoration[11]. Phylogenetic and population genomics data are vital for developing effective conservation and management strategies. With the rapid development of next-generation sequencing technology such as Illumina sequencing, chloroplast genome assembly has become less expensive and easier than it was with the Sanger method. Additionally, comparative analysis of the complete chloroplast genome among closely related species has proven to be a valid and effective method for the studying evolutionary history, species conservation, and phylogenetic relationships[12–15].

Chloroplasts are essential organelles in plant cells that play very important roles in photosynthesis, carbon fixation, and synthesis of pigments, starch, fatty acids, and amino acids[16, 17]. The chloroplast genome of angiosperms typically consists of highly conserved circular DNA ranging from 120 to 180 kb in length with a typical quadripartite structure including a large single-copy (LSC) region, a small single-copy (SSC) region, and a pair of inverted repeats (IRs)[18]. The chloroplast genome encodes approximately 110 to 130 genes, including up to 80 unique protein-coding genes, four ribosomal RNAs (rRNAs), and approximately 30 transfer RNAs (tRNAs)[19]. In recent years, many complete chloroplast genome sequences of higher plants have been reported and used to study population structure and phylogenetic relationships.

In this study, we sequenced the *C*. *sclerophylla* chloroplast genome using Illumina technology. This is the first comprehensive analysis of the *C*. *sclerophylla* chloroplast genome in conjunction with the previously published whole-chloroplast genome sequences of three congeneric species. In addition, we used 22 complete chloroplast genome sequences from GenBank to analyze phylogenetic relationships and infer the phylogenetic position of *C*. *sclerophylla*. The results not only provide basic knowledge about the characteristics of *C*. *sclerophylla* but also enhance our understanding of *Castanopsis* species evolution within the family Fagaceae. Our data will contribute to our understanding of the genetic resources and evolution of *C*. *sclerophylla* based on the diversity in its chloroplast genome and also facilitate the exploration, utilization and application of conservation genetics of this species.

## Materials and methods

### Plant material, DNA extraction and sequencing

Fresh young leaves of *C*. *sclerophylla* were collected from the Jiangxi Agricultural University Arboretum in Nanchang, China (28°45'N, 115°49'E). Total genomic DNA was extracted using a Plant Genomic DNA Kit (TIANGEN, Beijing, China). Agarose gel electrophoresis and a microplate spectrophotometer (Molecular Device, Sunnyvale, CA, USA) were used to measure DNA quality and concentration, respectively. Shotgun libraries with an average insert size of 350 bp were constructed using pure DNA and sequenced from 150 bp paired-end read lengths with the Illumina HiSeq 2500 platform (Illumina, San Diego, California, USA). To obtain high-quality clean data, raw reads were filtered by removing the connector sequence and low-quality reads using NGS QC Toolkit_v.2.3.3[20].

### Chloroplast genome assembly and annotation

The high-throughput raw reads were trimmed by FastQC. Next, the trimmed paired-end reads and references (*C*. *hainanensis*, *C*. *echinocarpa*, and *C*. *concinna*) were used to extract chloroplast-like reads, which were assembled by NOVOPlasty[21]. NOVOPlasty assembled partial reads and stretched them as far as possible until a circular genome is formed. A high-quality complete chloroplast genome was ultimately obtained. The assembled genome was annotated using CpGAVAS[22]. BLAST and Dual Organellar Genome Annotator (DOGMA) wereapplied to check the annotation results[23]. tRNAs were identified by tRNAscan-SE[24]. Circular gene maps of *C*. *sclerophylla* were drawn with the OGDRAW v1.2 program[25]. To analyze variation in synonymous codon usage, MEGA7 was used to compute relative synonymous codon usage (RSCU) values, codon usage, and GC content[26]. RSCU represent the ratio of the observed frequency of a codon to the expected frequency and is a good indicator of codon usage bias[27]. When the RSCU value is less than 1, synonymous codons are used less frequently than expected; otherwise, the value is greater than 1[28].

### Comparative analysis and phylogenetic analysis

MUMmer[29] was employed for paired sequence alignment of the chloroplast genomes. Sequence divergence was computed pairwise distance between each two species adopting protein-coding sequences using MEGA 5.0 with Kimura 2-parameter model[30]. The mVISTA[31] program was used to compare the complete chloroplast genome of *C*. *sclerophylla* to three other published chloroplast genomes of the genus *Castanopsis*, i.e., *Castanopsis concinna* voucher Strijk_1489 (KT793041.1), *C*. *echinocarpa* (KJ001129.1), and *C*. *hainanensis* (MG383644.1), in Shuffle-LAGAN mode, adopting the annotation of *C*. *concinna* as a reference.

In total, 20 chloroplast genomes belonging to Fagaceae were analyzed in this study, including the newly generated chloroplast genome *C*. *sclerophylla* and all of the published chloroplast genomes (data present in NCBI GenBank on 31.12.2018). The other 19 chloroplast (cp) genomes species are from the specises *Castanea henryi* voucher CHEN20160703 (KX954615.1), *Castanea mollissima* (HQ336406.1), *Castanopsis concinna* voucher Strijk_1489 (KT793041.1), *Castanopsis echinocarpa* (KJ001129.1), *Castanopsis hainanensis* (MG383644.1), *Fagus engleriana* (KX852398.1), *Lithocarpus balansae* (KP299291.1), *Quercus aliena* (KP301144.1), *Quercus aquifolioides* (KP340971.1), *Quercus baronii* (KT963087.1), *Quercus dolicholepis* (KU240010.1), *Quercus glauca* (KX852399.1), *Quercus rubra* (JX970937.1), *Quercus sichourensis* (MF787253.1), *Quercus spinosa* (KM841421.1), *Quercus tarokoensis* (MF135621.1), *Quercus tungmaiensis* (MF593893.1), *Quercus variabilis* (KU240009.1), and *Trigonobalanus doichangensis* (KF990556.1). Phylogenies were constructed by maximum likelihood (ML) using the 20 cp genomes of the Fagaceae species in GenBank. *Corylus fargesii* (KX822767.2) and *Eucalyptus umbra* (KC180778.1) were used as outgroups. Sequences were initially aligned using MAFFT[32], followed by visualization and manual adjustment of multiple sequence alignment in BioEdit[33]. The maximum likelihood (ML) analysis was conducted using RAxML web servers[34]. For ML analyses, general time reversible (GTR)+ G model was used in as suggested by 1,000 bootstrap replicates with the default tree search algorithm of hill-climbing[30, 35, 36].

## Results and discussion

### Characteristics of *C. sclerophylla* cpDNA

**Fig 1:**
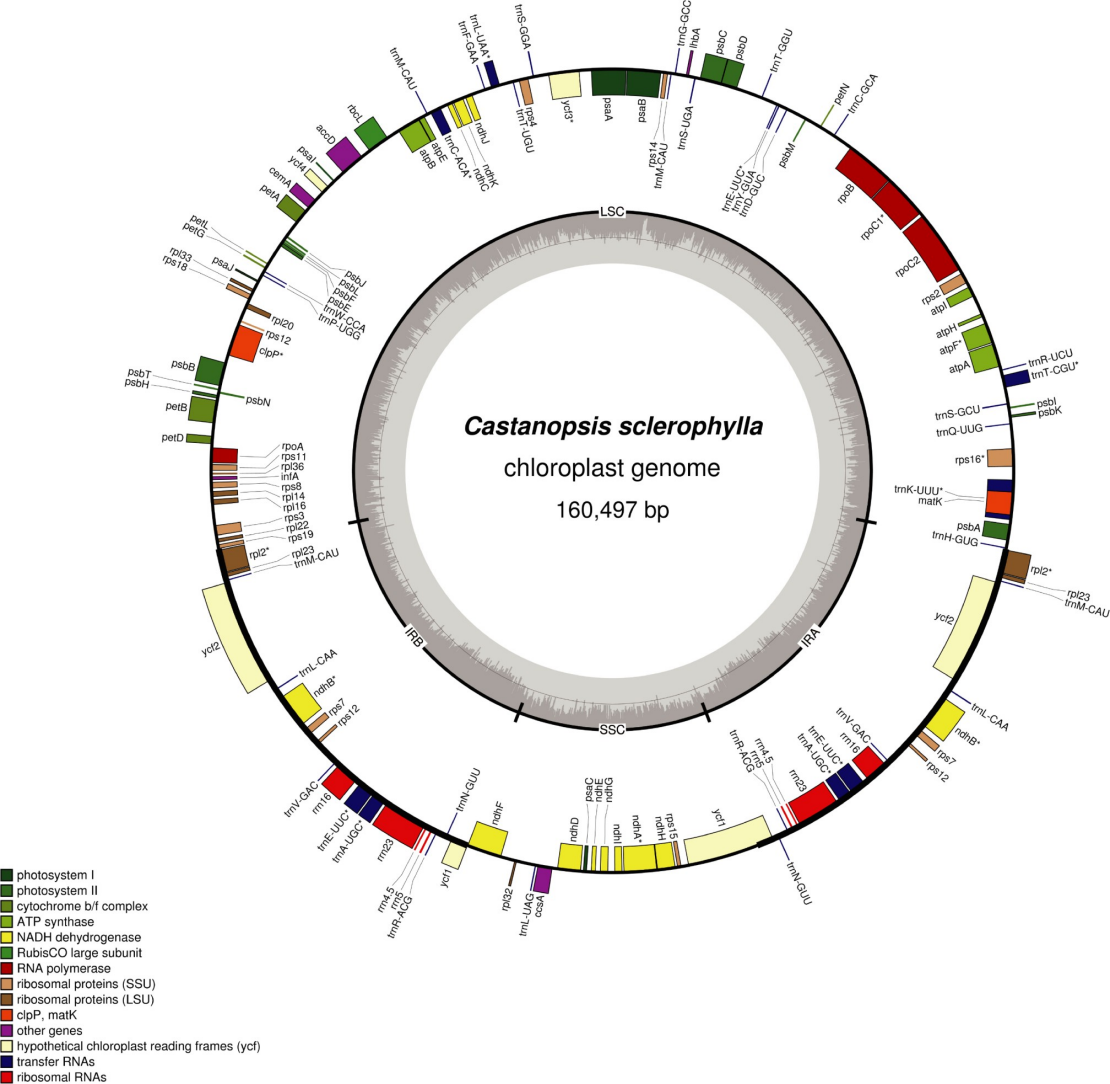
Chloroplast genome annotation map for *C*. *sclerophylla*. Genes inside the circle are transcribed in a clockwise direction; genes outside are transcribed in a counterclockwise direction. Different colors represent different functional genes. The darker gray and lighter gray in the inner circle show the GC and AT contents of the chloroplast genome, respectively.

A total of 65 million paired-end reads were obtained, and 10.44 Gb of high-quality clean data with a mean Q30 higher than 88.28% were obtained by removing low-quality reads and connector sequence. The remaining high-quality reads were utilized in the further assembly. The complete chloroplast genome sequence of *C*. *sclerophylla* is 160,497 bp in length; it has been deposited in GenBank under accession number MK387847. The genome has a typical quadripartite structure including a pair of IR (IRa and IRb) regions of 25,675 bp that are separated by an LSC region of 90,255 bp and an SSC region of 18,892 bp (Fig 1, Table 1). The overall GC content of the chloroplast genome is 36.82%, which is similar to that of other Fagaceae species[37–39]. However, a few differences in GC content were found among the chloroplast genomes. The GC contents of the LSC, SSC, and IR regions are 34.65%, 30.94%, and 42.78%, respectively (Table 2). The GC content is highest in IR regions (42.78%), likely due to the presence of four duplicated ribosomal RNA genes in this region, a pattern also found in the chloroplast genome of *C*. *hainanensis*[38]. The overall GC content is an important species indicator[40].

**Table 1:** Summary of the characteristics of four *Castanopsis* chloroplast genomes.

Genome	*C*. *sclerophylla*	*C*. *hainanensis*	*C*. *echinocarpa*	*C*. *concinna*
**Genome size (bp)**	*160*,*497*	160,631	160,647	160,606
**LSC length (bp)**	90,255	90,328	90,394	90,368
**SSC length (bp)**	18,892	18,929	18,995	18,884
**IR length (bp)**	25,675	25,687	25,629	25,677
**Number of genes**	131	132	132	136
**Number of** **protein-coding genes**	86	84	84	82
**Number of tRNA genes**	37	40	40	46
**Number of rRNA genes**	8	8	8	8

**Table 2:** Base content of the *C*. *sclerophylla* chloroplast genome.

Region	A (%)	T (%)	C (%)	G (%)	A+T (%)	G+C (%)
**LSC**	31.94	33.4	17.74	16.91	65.34	34.65
**SSC**	34.4	34.66	16.29	14.65	69.06	30.94
**IR**	28.61	28.61	21.39	21.39	57.22	42.78
**Total**	31.65	32.23	18.47	17.65	63.18	36.82

A total of 131 genes were found in the *C*. *sclerophylla* chloroplast genome, including 86 protein-coding genes, 37 tRNA genes, and 8 rRNA genes (Fig 1, Table 1). Of these 131 genes, 110 genes are unique and annotated and divided into three categories: 79 protein-coding genes, 27 tRNA genes, and four rRNA genes (Table 3). In addition, 21 functional genes (seven protein-coding genes, four rRNA genes, and 10 tRNA genes) are duplicated in the IR regions (Fig 1). The LSC region comprises 62 protein-coding genes and 22 tRNA genes, whereas the SSC region comprises 11 protein-coding and one tRNA gene (S1 Table). There are 14 intron-containing genes, including eight protein-coding genes and six tRNA genes. Twelve genes contain one intron,and *clpP* and *ycf3* have two introns. *trnK-UUU* contains the longest intron (2,511 bp); and *trnL-UAA* the shortest (485 bp) (Table 4). A similar phenomenon is also present in *Quercus acutissima*[41]. *ycf3* gene expression results in stable accumulation of photosystem I complexes [42]. Therefore, we herein focus on the *ycf3* intron gain in *C*. *sclerophylla*, which may be helpful for further study of the photosynthesis mechanism.

**Table 3:** List of genes annotated in the sequenced *C*. *sclerophylla* chloroplast genome.

Category	Function	Genes
**Photosynthesis**	Photosystem I	*psaA*, *psaB*, *psaC*, *psaI*, *psaJ*
	Photosystem II	*psbA*, *psbB*, *psbC*, *psbD*, *psbE*, *psbF*, *psbH*, *psbI*, *psbJ*, *psbK*, *psbL*, *psbM*, *psbN*, *psbT*
	Cytochrome b/f complex	*petA*, *petB*, *petD*, *petG*, *petL*, *petN*
	ATP synthase	*atpA*, atpB, *atpE*, atpF*, *atpH*, *atpI*
	NADH dehydrogenase	ndhA*, ndhB*(X2), *ndhC*, *ndhD*, *ndhE*, *ndhF*, *ndhG*, *ndhH*, *ndhI*, *ndhJ*, *ndhK*
	Rubisco large subunit	*rbcL*
**Self-replication**	RNA polymerase	rpoA, rpoB, rpoC1*, rpoC2
	Ribosomal proteins (LSU)	rpl14, rpl16, rpl2*(X2), rpl20, rpl22, rpl23(X2), rpl32, rpl33, rpl36
	Ribosomal proteins (SSU)	rps11, rps12(X2), rps14, rps15, rps16*, rps18, rps19, rps2, rps3, rps4, rps7(X2), rps8
	Transfer RNAs	trnA-UGC*(X2), trnC-ACA*, *trnC-GCA*, *trnD-GUC*, trnE-UUC*(X3), *trnF-GAA*, *trnG-GCC*, *trnH-GUG*, trnK-UUU*, *trnL-CAA*(X2), *trnL-CAA*, trnL-UAA*, *trnL-UAG*, *trnM-CAU*(X4), *trnN-GUU*(X2), *trnP-UGG*, *trnQ-UUG*, *trnR-ACG*(X2), *trnR-UCU*, *trnS-GCU*, *trnS-GGA*, *trnS-UGA*, trnT-CGU*, *trnT-GGU*, *trnT-UGU*, *trnV-GAC*(X2), *trnV-GAC*, *trnW-CCA*, *trnY-GUA*
	Ribosomal RNAs	*rrn16*, *rrn23*, *rrn4*.*5*, *rrn5*
**Others**	Hypothetical chloroplast reading frames	*ycf1*(X2), *ycf2*(X2), ycf3**, *ycf4*
	Other genes	*ccsA*, c*emA*, clpP**, *infA*, *lhbA*, *matK*

(×n) number of gene copies in the IR.

* Genes containing one intron

** genes containing two introns.

**Table 4:** Lengths of exons and introns for genes with introns in the *C*. *sclerophylla* chloroplast genome.

Gene	Location	Exon I (bp)	Intron I (bp)	Exon II (bp)	Intron II (bp)	Exon III (bp)
***clpP***	LSC	70	851	291	654	227
***trnK-UUU***	LSC	36	2511	34		
***rpoC1***	LSC	429	838	1618		
***trnC-ACA***	LSC	37	610	55		
***ndhB***	IRA	776	681	755		
***ndhA***	SSC	550	1049	540		
***rpl2***	IRB	390	685	433		
***trnA-UGC***	IRB	36	801	35		
***trnL-UAA***	LSC	34	485	49		
***trnE-UUC***	LSC	31	956	39		
***trnT-CGU***	LSC	34	720	42		
***rps16***	LSC	41	903	227		
***ycf3***	LSC	125	727	225	768	154
***atpF***	LSC	144	789	409		

### Codon usage analysis

**Fig 2:**
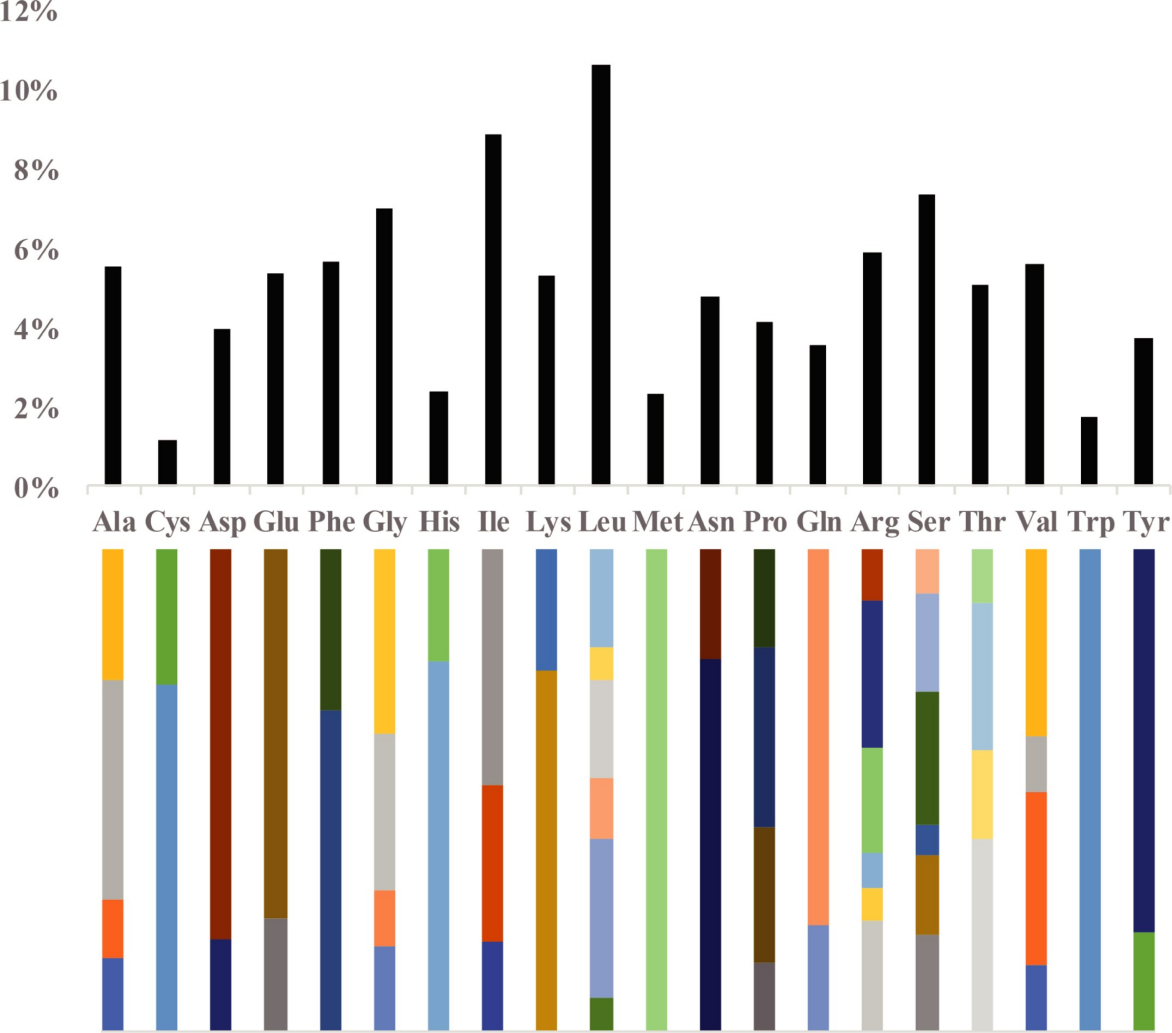
Codon numbers of twenty kinds of amino acids and stop codon of protein-coding sequences for the *C*. *sclerophylla* chloroplast genome. Different colors of the histogram represent the proportion of codon usage and stop codon.

Relative synonymous codon usage frequency (RSCU) valueswere computed for the *C*. *sclerophylla* chloroplast genome using protein-coding sequences (S2 Table), as codon usage plays a vital role in shaping chloroplast genome evolution[43]. In total, 23,131 codons are present. Leucine (10.61%) is the most commonly encoded amino acid, with 2,454 codons, followed by isoleucine (8.85%) with 2048 codons; cysteine (1.13%) is the least commonly encoded amino acid, with 262 codons (Fig 2). Similar ratios for amino acids were previously reported for chloroplast genomes[44, 45]. Moreover, methionine and tryptophan are encoded by only one codon, indicating no codon bias for these two amino acids (RSCU = 1). Nearly all of the codons ending with A and U had RSCU values of more than one (RSCU > 1), whereas the codons ending with C and G had RSCU values of less than one. The AU contents for the first, second, and third codon positions were calculated to be 54.07%, 56.29% and 70.20%, respectively. The results of high AU content at the third codon position were similar to reports for other plants[46].

### Comparative analysis of genomic structure

**Fig 3:**
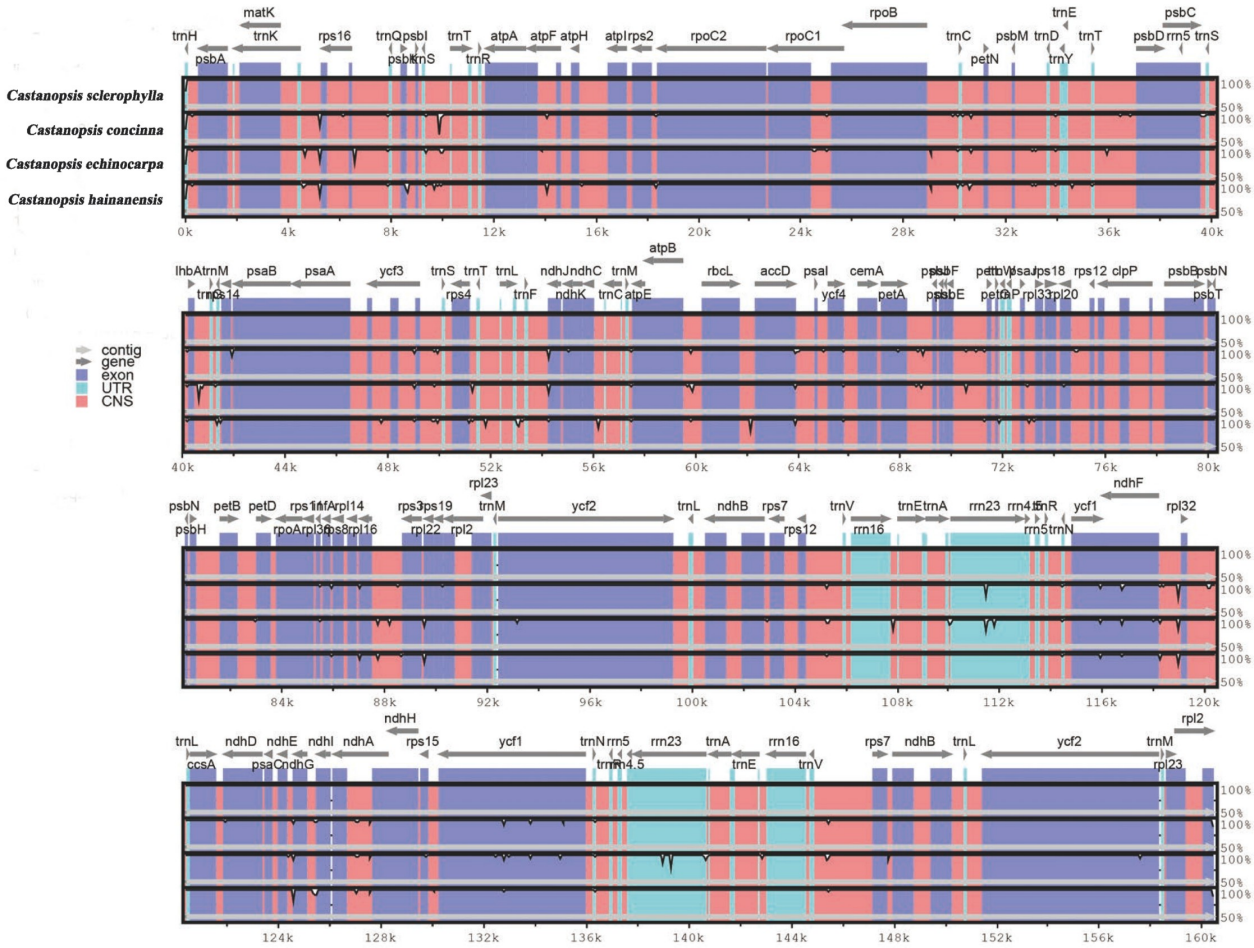
Visualization of alignment of the complete chloroplast genome of four species by the program mVISTA using *C*. *concinna* as a reference. The gray arrows and thick black lines above the alignment indicate the orientation of genes. Blue bars represent exons, sky-blue bars represent untranslated regions (UTRs), and pink bars represent noncoding sequences (NCSs). The vertical scale represents the percent identity within 50–100%.

Three complete chloroplast genomes within the *Castanopsis* genus (*C*. *hainanensis*, *C*. *echinocarpa*, and *C*. *concinna*) were selected for comparison with that of *C*. *sclerophylla*. *C*. *sclerophylla* has the smallest chloroplast genome (160,497 bp); *C*. *echinocarpa* has the largest chloroplast genome (160,647 bp) with the smallest IR region (25,629 bp). Additionally, the lengths of LSC regions varied among these four species, from 90,255 bp in *C*. *sclerophylla* to 90,394 bp in *C*. *echinocarpa* (Table 1). The different lengths of the LSC region are the main reason for the difference in sequence length among the four species, consistent with the results for the genus *Oryza*[44]. To investigate levels of genome divergence, the program mVISTA was used to plot sequence identity for the chloroplast genomes of the four species using *C*. *concinna* as a reference (Fig 3). The results of sequence analysis revealed the LSC and SSC regions to be more divergent than the IR regions among the four *Castanopsis* genomes; furthermore, greater divergence was found in noncoding regions than in coding regions. Coding regions with significant variation in the four chloroplast genomes included *ndhF*, *ndhG*, and *ycf1*, which are all located in SSC regions. Nonetheless, the most divergent regions were observed in intergenic regions, including *trnK*-*rps16*, *trnS*-*trnT*, *atpA*-*atpF*, *trnC*-*petN*, *trnT*-*psbD*, *IhbA*-*trnG*, *ycf3*-*trnS*, *rps4*-*trnT*, *trnT*-*trnL*, *atpB*-*rbcL*, *petA*-*psbJ*, *psbE*-*petL*, *rpl16*-*rpl3*, and *ndhF*-*rpl32*.

**Fig 4:**
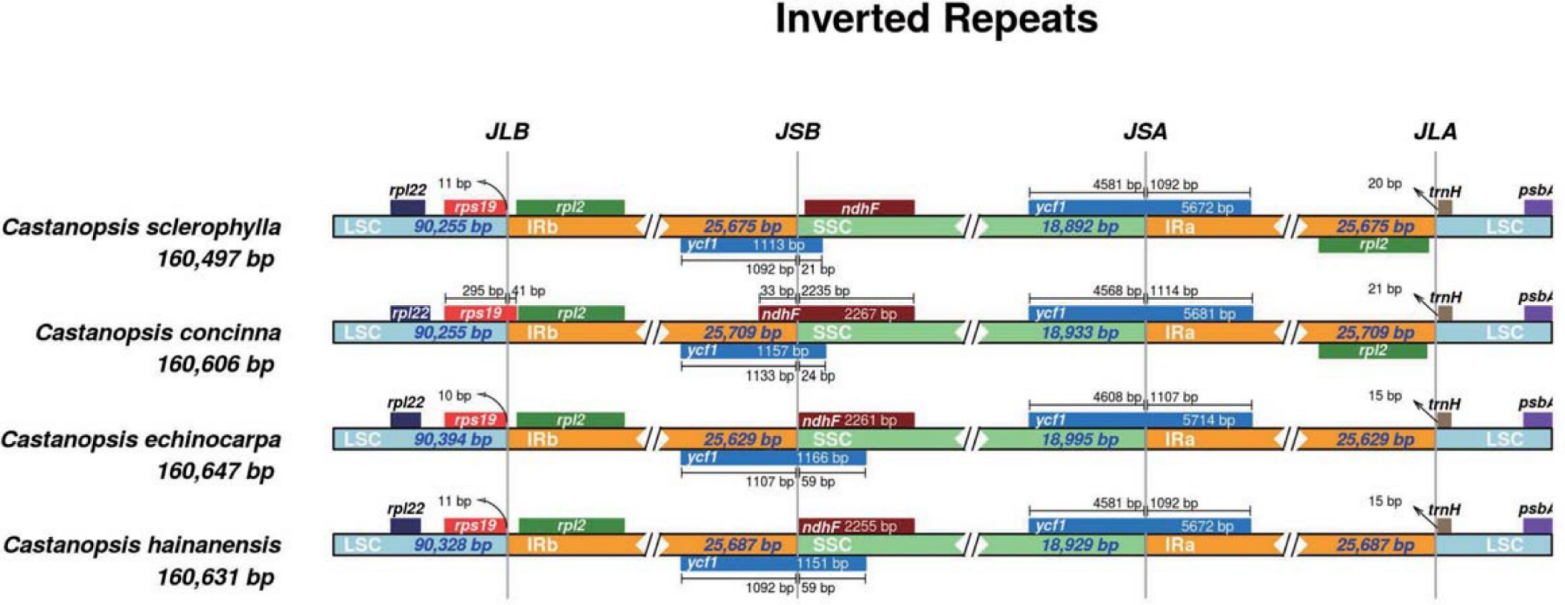
Comparison of junctions of large single-copy (LSC), small single-copy (SSC), and inverted repeat (IR) regions among the chloroplast genomes of four congeneric species. The genes transcribed on the positive strand are depicted on the top of their corresponding locus from right to left; negative strand genes are depicted below from left to right. The arrows indicate the distance between the start or end of a given gene and the corresponding junction site. JLB (LSC/IRb), JSB (IRb/SSC), JSA (SSC/IRa) and JLA (IRa/LSC) denote four junctions in the genome between the two single-copy regions (LSC and SSC) and the two IRs (IRa and IRb).

The expansion and contraction of IR regions at the borders are the major reason for chloroplast genome size variation and play vital roles in evolution[47–49]. A detailed comparison of four junctions (JLA, JSB, JSA, and JLA) between the two single-copy regions (LSC and SSC) and the two IRs (IRa and IRb) was performed for *C*. *sclerophylla*, *C*. *hainanensis*, *C*. *echinocarpa* and *C*. *concinna* by analyzing exact IR border positions and adjacent genes (Fig 4). Overall IR regions are relatively conserved in the genus *Castanopsis*, and this result agrees with reports for the genus *Quercus*[41]. The *rpsl9* gene is located between the junction of the LSC and IRb regions in *C*. *concinna*. However, in the *C*. *sclerophylla*, *C*. *hainanensis*, and *C*. *echinocarpa* chloroplast genomes, the *rps19* gene is located in the LSC region and is 11 bp, 11 bp, and 10 bp from the border of the LSC region, respectively. Some studies have indicated that *ycf1* is required for plant viability and encodes Tic214, which is a vital component of the TIC complex in *Arabidopsis*[50, 51]. The *ycf1* gene crosses the SSC/IRb and SSC/IRa regions. The SSC/IRb junction is located in the *ycf1* region in the chloroplast genome of all four *Castanopsis* species and extends into the SSC region by different lengths depending on the genome (*C*. *sclerophylla*, 21 bp; *C*. *hainanensi****s***, 24 bp; *C*. *echinocarpa*, 59 bp; and *C*. *concinna*, 59 bp); the IRb region includes 1,131, 1,157, 1,107, and 1,092 bp of the *ycf1* gene. The SSC/IRa junction also extends into the SSC region by different lengths depending on the genome (*C*. *sclerophylla*, 4,581 bp; *C*. *hainanensis*, 4,568 bp; *C*. *echinocarpa*, 4,608 bp; and *C*. *concinna*, 4,581 bp); the IRa region includes 1,092, 1,114, 1,107, and 1,092 bp of the *ycf1* gene.

### Phylogenetic analysis

**Fig 5:**
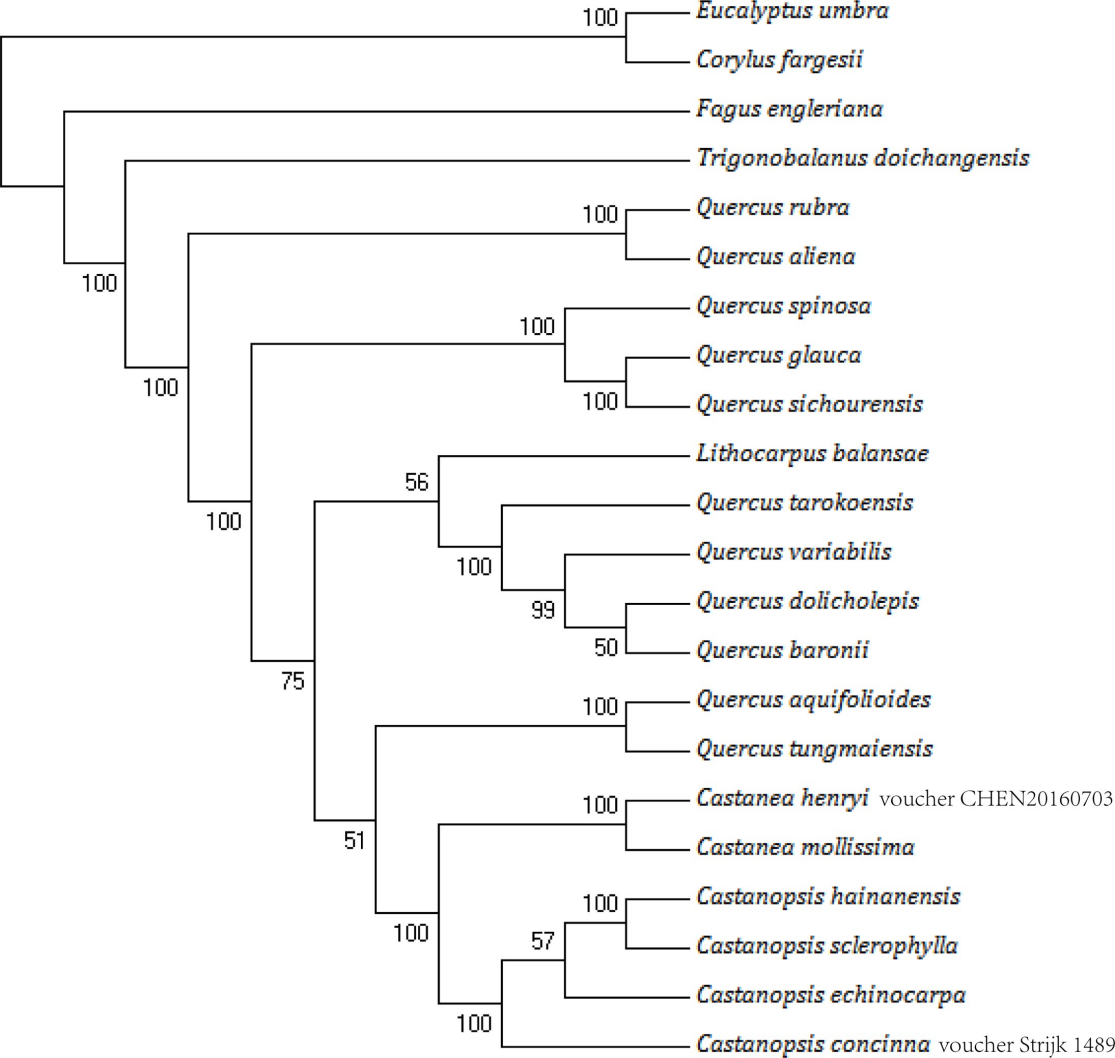
A maximum likelihood (ML) phylogenetic tree was constructed based on the chloroplast genomes of 22 species. *C*. *fargesii* and *E*. *umbra* were used as outgroups.

Phylogenetic analysis was performed by ML based on the 22 aligned sequences of chloroplast genomes (Fig 5). *C*. *fargesii* and *E*. *umbra* were used as outgroups. The ML-based phylogenetic analysis showed that these four species of the genus *Castanopsis* form a monophyletic clade and that *C*. *sclerophylla* is closely related *to* C. hainanensis with strong bootstrap values. The ML tree indicated that *Castanopsis* is closely related to *Castanea*. Surprisingly, *Quercus* species do not form a clade, and *Quercus* is not divided into two clusters containing either evergreen or deciduous tree species. The phylogenetic status of these genera is consistent with a previous report[41, 52, 53]. The relatively high variation in *Quercus* may be related to the widely distributed range which need to local adaptation to different environments. Notably, *F*. *engleriana* is the first to diverge in Fagaceae, which indicates the relatively high genetic divergence between *F*. *engleriana* and others, followed by *T*. *doichangensis*, which indicates that they are early diverging taxa in Fagaceae[54]. Moreover, the same topology results of genus *Fagus* was confirmed by the research based on nuclear marker[55].

Little is known to date about the chloroplast genome of *Castanopsis*, and only three chloroplast genome sequences of *Castanopsis* species can be found in GenBank, which has greatly hampered the study of the phylogenetic relationships of this genus. Therefore, more research on the complete chloroplast genomes of *Castanopsis* species needs to be conducted in the future.

## Conclusions

*C*. *sclerophylla* is an important evergreen broad-leaved species in the *Castanopsis* genus of the Fagaceae family. In this study, the complete chloroplast genome sequence of *C*. *sclerophylla* was determined using the Illumina HiSeq 2500 platform. The *C*. *sclerophylla* chloroplast genome exhibits a typical quadripartite and circular structure similar to that of the chloroplast genome of three congeneric species. Compared to the chloroplast genomes of the three other *Castanopsis* species, that of *C*. *sclerophylla* is the smallest (160,497 bp). In the ML phylogenetic tree, the phylogenetic relationships among 22 angiosperms strongly support the known classification of *C*. *sclerophylla*, and ML analysis showed that these four *Castanopsis* species form a monophyletic clade and that *C*. *sclerophylla* is closely related to *C*. *hainanensis* with strong bootstrap values. In addition, *Castanopsis* is closely related to *Castanea*. The genus *Castanopsis* contains approximately 120 known species, nearly half of which are native to China. Indeed, China has a large amount of *Castanopsis* germplasm resources, and the availability of chloroplast genomes provides a powerful genetic resource for phylogenetic analysis and biological study. Therefore, further research of the complete chloroplast genome of the genus *Castanopsis* is necessary in the future. The data will contribute to the development of genetic resources and the identification of evolutionary relationships and also facilitate the exploration, utilization and application of conservation genetics for the genus.

## Supporting information

S1 Table The number of genes in the *C*. *sclerophylla* chloroplast genome. (DOCX)

S2 Table Codon–anticodon recognition pattern and codon usage for the *C*. *sclerophylla* chloroplast genome. (DOCX)
