# Supplementary material for: Complete chloroplast genome of Castanopsis sclerophylla (Lindl.) Schott: Genome structure and comparative and phylogenetic analysis
Source: PLoS One. 2019 Jul 30;14(7):e0212325. doi: 10.1371/journal.pone.0212325 (PMC6667119; doi:10.1371/journal.pone.0212325)
Supplement: S1 Table — (DOCX) [file pone.0212325.s001.docx]

# S1 Table

# S1 Table The number of genes in the *C. sclerophylla* chloroplast genome

| **Region** | **Number of CDSs** | **Number of tRNAs** | **Number of rRNAs** | **Total** |
| --- | --- | --- | --- | --- |
| **LSC region** | 62 | 22 | 0 | 84 |
| **SSC region** | 11 | 1 | 0 | 12 |
| **IRa region** | 7 | 7 | 4 | 18 |
| **IRb region** | 6 | 7 | 4 | 17 |
