# Supplementary material for: Complete chloroplast genome of Castanopsis sclerophylla (Lindl.) Schott: Genome structure and comparative and phylogenetic analysis
Source: PLoS One. 2019 Jul 30;14(7):e0212325. doi: 10.1371/journal.pone.0212325 (PMC6667119; doi:10.1371/journal.pone.0212325)
Supplement: S2 Table — (DOCX) [file pone.0212325.s002.docx]

# S2 Table

**S2 Table Codon–anticodon recognition pattern and codon usage for the *C. sclerophylla* chloroplast genome.**

| **Amino acids** | **Codon** | **No.** | **RSCU** | **tRNA** | **Amino acids** | **Codon** | **No.** | **RSCU** | **tRNA** |
| --- | --- | --- | --- | --- | --- | --- | --- | --- | --- |
| **Ala** | GCC | 196 | 0.61 |  | Asn | AAU | 858 | 1.55 |  |
| **Ala** | GCG | 158 | 0.49 |  | Asn | AAC | 250 | 0.45 |  |
| **Ala** | GCU | 581 | 1.81 | *trnA-UGC* | Pro | CCG | 136 | 0.56 |  |
| **Ala** | GCA | 348 | 1.08 |  | Pro | CCA | 270 | 1.12 |  |
| **Cys** | UGU | 189 | 1.44 | *trnC-ACA* | Pro | CCU | 362 | 1.5 | *trnP-UGG* |
| **Cys** | UGC | 73 | 0.56 | *trnC-GCA* | Pro | CCC | 195 | 0.81 |  |
| **Asp** | GAC | 176 | 0.38 |  | Gln | CAG | 179 | 0.44 |  |
| **Asp** | GAU | 740 | 1.62 |  | Gln | CAA | 638 | 1.56 |  |
| **Glu** | GAG | 291 | 0.47 |  | Arg | CGA | 312 | 1.38 |  |
| **Glu** | GAA | 950 | 1.53 |  | Arg | CGC | 94 | 0.41 |  |
| **Phe** | UUU | 876 | 1.33 | *trnL-UAA* | Arg | CGG | 99 | 0.44 |  |
| **Phe** | UUC | 438 | 0.67 | *trnN-GUU* | Arg | CGU | 293 | 1.29 | *trnR-ACG* |
| **Gly** | GGG | 282 | 0.7 |  | Arg | AGA | 418 | 1.84 |  |
| **Gly** | GGC | 191 | 0.47 | *trnG-GCC* | Arg | AGG | 145 | 0.64 |  |
| **Gly** | GGU | 526 | 1.3 |  | Ser | UCA | 339 | 1.2 |  |
| **Gly** | GGA | 615 | 1.52 |  | Ser | UCC | 285 | 1 | *trnS-GGA* |
| **His** | CAU | 419 | 1.54 |  | Ser | AGC | 104 | 0.37 |  |
| **His** | CAC | 126 | 0.46 |  | Ser | UCU | 474 | 1.67 | *trnT-UGU* |
| **Ile** | AUC | 384 | 0.56 |  | Ser | AGU | 345 | 1.22 |  |
| **Ile** | AUA | 664 | 0.97 |  | Ser | UCG | 155 | 0.55 | *trnT-CGU* |
| **Ile** | AUU | 1000 | 1.46 |  | Thr | ACU | 474 | 1.61 |  |
| **Lys** | AAA | 920 | 1.5 |  | Thr | ACC | 217 | 0.74 |  |
| **Lys** | AAG | 306 | 0.5 |  | Thr | ACA | 355 | 1.2 |  |
| **Leu** | CUG | 170 | 0.42 |  | Thr | ACG | 133 | 0.45 |  |
| **Leu** | UUA | 810 | 1.98 |  | Val | GUG | 180 | 0.56 |  |
| **Leu** | CUA | 316 | 0.77 |  | Val | GUU | 461 | 1.43 | *trnE-UUC* |
| **Leu** | CUU | 496 | 1.21 | *trnL-UAG* | Val | GUC | 154 | 0.48 | *trnV-GAC* |
| **Leu** | CUC | 170 | 0.42 | *trnH-GUG* | Val | GUA | 499 | 1.54 |  |
| **Leu** | UUG | 492 | 1.2 | *trnM-CAU* | Trp | UGG | 402 | 1 | *trnW-CCA* |
| **Met** | AUG | 533 | 1 |  | Tyr | UAC | 179 | 0.41 |  |
|  |  |  |  |  | Tyr | UAU | 690 | 1.59 |  |

Note: RSCU indicates relative synonymous codon usage.
